# Developing SysteMatic: Prevention, precision and equity by design for people living with multiple long-term conditions

**DOI:** 10.1177/26335565241272682

**Published:** 2024-09-30

**Authors:** Frances S. Mair, Farnaz Nickpour, Barbara Nicholl, Sara MacDonald, Dan W. Joyce, Jonathan Cooper, Nic Dickson, Isobel Leason, Qammer H. Abbasi, Izzettin F. Akin, Fani Deligianni, Elizabeth Camacho, Jennifer Downing, Hilary Garrett, Martina Johnston Gray, David J. Lowe, Muhammad A. Imran, Sandosh Padmanabhan, Colin McCowan, P. John Clarkson, Lauren E. Walker, Iain Buchan

**Affiliations:** 1 General Practice and Primary Care, School of Health and Wellbeing, 3526 University of Glasgow, Glasgow, UK; 2 The Inclusionaries Lab for Design Research, School of Engineering, 4591 University of Liverpool, Liverpool, UK; 3 Institute of Population Health, 4591 University of Liverpool, Liverpool, UK; 4 James Watt School of Engineering, 3526 University of Glasgow, Glasgow, UK; 5 School of Engineering, 4591 University of Liverpool, Liverpool, UK; 6 School of Computing Science, 3526 University of Glasgow, Glasgow, UK; 7 Department of Primary Care and Mental Health, 4591 University of Liverpool, Liverpool, UK; 8 NIHR ARC NWC, 4591 University of Liverpool, UK; 9 Public Advisor, North-West Glasgow Voluntary Sector Network SCIO, Glasgow, UK; 10 Emergency Department, Queen Elizabeth University Hospital, Glasgow, UK; 11 BHF Glasgow Cardiovascular Research Centre, School of Cardiovascular and Metabolic Health, 3526 University of Glasgow, Glasgow, UK; 12 School of Medicine, 7486 University of St Andrews, St Andrews, UK; 13 Department of Engineering, 2152 University of Cambridge, Cambridge, UK; 14 Centre for Experimental Therapeutics, 4591 University of Liverpool, Liverpool, UK

**Keywords:** Multimorbidity, digital health, health technology, wearable devices

## Abstract

**Background:**

The number of individuals living with multiple (≥2) long term conditions (MLTCs) is a growing global challenge. People with MLTCs experience reduced life expectancy, complex healthcare needs, higher healthcare utilisation, increased burden of treatment, poorer quality of life and higher mortality. Evolving technologies including artificial intelligence (AI) could address some of these challenges by enabling more preventive and better integrated care, however, they may also exacerbate inequities.

**Objective:**

We aim to deliver an equity focused, action-ready plan for transforming MLTC prevention and care, co-designed by people with lived experience of MLTCs and delivered through an Innovation Hub: SysteMatic.

**Design:**

Our Hub is being co-designed by people with lived experience of MLTCs, practitioners, academics and industry partners in Liverpool and Glasgow, UK. This work builds on research into mental-physical health interdependence across the life-course, and on mobilisation of large-scale quantitative data and technology validation in health and care systems serving deprived populations in Glasgow and Liverpool. We work with 3 population segments: 1) Children & Families: facing psychosocial and environmental challenges with lifetime impacts; 2). Working Life: people with poorly integrated mental, physical and social care; and 3) Pre-Frailty: older people with MLTCs. We aim to understand their experiences and in parallel look at routinely collected health data on people with MLTCs to help us identify targets for intervention. We are co-identifying opportunities for systems transformation with our patient partners, healthcare professionals and through discussion with companies and public-sector organisations. We are co-defining 3/5/7-year MLTC innovation/transition targets and sustainable learning approaches**.**

**Discussion:**

SysteMatic will deliver an actionable MLTC Innovation Hub strategic plan, with investment from the UK National Health Service, civic health and care partners, universities, and industry, enabling feedback of well-translated, patient and public prioritised problems into the engineering, physical, health and social sciences to underpin future equitable innovation delivery.

## Background and rationale

Multiple long-term conditions (MLTCs) challenge patients, families and health systems.^1,2^ Individuals living with ≥2 long term conditions have reduced life expectancy, complex healthcare needs, higher healthcare utilisation, increased burden of treatment^3^ poorer quality of life^1,4^ and higher mortality.^5^ MLTCs are functions of interacting and multiple biological, lifestyle, social and environmental factors that vary over the life-course.^1^ MLTCs disproportionately affect more socioeconomically disadvantaged groups where prevalence is higher, and onset is around 10-15 years earlier.^6^ Combined mental and physical MLTCs are more prevalent in these populations^6^ and are associated with premature mortality (20-year lifespan reduction^7^) alongside reduced health-span (years lived in good health).^8,^^9^ Closing the mental-physical health divide and improving care pathways and coordination across health- and social-care systems is critical for meeting the needs of increasingly complex populations with MLTCs^1^ and the ability for healthcare systems to provide care within the context of rising demand and workforce challenges.

The prevention and management of MLTCs challenges traditional research and service models. For example, the usual approach to new technologies tends to focus on single diseases, ignoring common, real-world complexity. Digital transformation through e.g., remote consultation, remote monitoring (via wearable or remote, non-invasive sensing technologies), and the use of data-driven technologies including artificial intelligence (AI) and machine learning (ML) to support decision making, have potential to tackle the challenges posed by MLTCs. However, there remain large gaps in evidence and translation, with innovations failing to take root in services despite large investments.^10,11^ This failure of practical innovation can arise from narrow problem framing/solving, short implementation cycles, or from failing to examine the relationship between human actors and the wider context, which is vital for MLTC systems of care.

Co-creating, implementing, and embedding healthcare innovation involves complex processes of change. These changes are at the micro level for patients and professionals, at the meso level for providers, and at the macro level for commissioners and public health services.^10^

To avoid a one-size-fits-all over-simplification of MLTCs, it is vital to elicit and synthesise data on stakeholder-facing MLTC models (micro- and meso-scale system models) and compare them with the sometimes-conflicting priorities identified from macro-scale data.

Healthcare is delivered by different professionals across a range of settings and contexts, with various interconnections and interdependence making it a complex system (of professions and organisations providing care for a defined population). Therefore, in SysteMatic an interdisciplinary team of health, engineering, computing science, design, social science and arts-based researchers will employ systems engineering approaches^12,13^ and systems theory^14,15^ to pave the way for an Innovation Hub. SysteMatic will embed continuous learning of how to prevent MLTCs and improve care for affected individuals equitably, as core business of civic integrated health and care systems. We will work with stakeholders to define their *own* unmet needs and problem priorities for MLTC prevention and management using design-driven methodologies that embody participatory, inclusive, and human-centred design.

In this development phase, we will prepare SysteMatic for rapid scale-up, leveraging National Health Service (NHS)-embedded data and digital innovation hubs and MLTC research across the life-course in Liverpool and Glasgow, in the United Kingdom. The extreme socioeconomic disadvantage of our populations demands MLTC innovation that harnesses understanding of biopsychosocial determinants of MLTCs and diverse care contexts. Our pragmatic approach acknowledges that systems of MTLCs are complex, sparsely measured over the life-course, and need highly iterative and agile design and engineering of solutions. SysteMatic will therefore focus on three population segments in which our health and care systems are driving projects as catalysts for MLTC transformation. Our patient and public involvement and engagement (PPIE) groups advised us to focus on these segments and have shaped our programme of research.

### Aims and objectives

We aim to deliver an action-ready plan for transforming MLTC prevention and care through an Innovation Hub: SysteMatic. Our Hub will be co-designed by people with lived experience of MLTCs, healthcare practitioners, academics and industry partners in Liverpool and Glasgow. Planning will build on research into mental-physical health interdependence across the life-course, and on mobilisation of large-scale quantitative data and technology validation in health and care systems that serve deprived populations in Liverpool and Glasgow. We will produce an action ready plan and *initiate* SysteMatic through a 5-stage approach that is currently underway:

**Stage 0. Convene** a **design cooperative** of people with MLTC lived experience, health and care professionals, scientists, engineers and designers, to embed MLTC data-action-research and design (systems definition, optimisation, innovation and transition) as core business of health and care systems.

**Stage 1.** Map out MLTC intelligence in 3 key segments of the population:

i. **Children & Families**: facing psychosocial and environmental challenges with lifetime impacts.
ii. **Working Life**: with poorly integrated mental, physical and social care.
iii. **Pre-Frailty**: people with MLTCs and opportunities for pre-frailty interventions.

**Stage 2.** Co-identify opportunities for systems transformation using the intelligence from stage 1, and further discussion with companies and public-sector organisations developing new technologies for person-centred complex care and population health management.

**Stage 3.** Co-define 3/5/7-year MLTC innovation/transition targets and sustainable learning approaches. PPIE-ranked targets can draw on our innovation pipelines, with prototypes, for example, of ambient sensing (that capture health data continuously and unobtrusively within a given physical environment without having to wear a device), and wearable sensing (for example using a smart watch or medical device) for managed self-care and earlier comorbidity diagnosis, proactive decision-making and better/shared care.

**Stage 4.** Co-produce a MLTC Innovation Hub strategic plan**,** with investment from the NHS, civic health and care partners, universities and industry, enabling feedback of well-translated, PPIE-prioritised problems into the engineering and design, physical, health and social sciences.

## Developing an innovation hub through a design cooperative

### Vision and General Approach (Figure 1)

We will design SysteMatic through 3 working groups studying **systems**, **people** and **intelligence**, in a cooperative of: those living with MLTCs, health and care professionals, scientists, engineers and designers. We will apply design rigour and use large-scale quantitative and rich qualitative data to produce MLTC intelligence arrays (e.g. visualisations describing multiple condition morbidity patterns across geographies), systems failure scenarios (e.g. where stakeholders identify how and when existing healthcare systems fail for managing their conditions), and when possible, identify and map these outputs to relevant system theory. The working groups will converge in workshops to: 1) co-define and co-prioritise problems, system elements and failure points, complexities and interdependencies, and stakeholder relations and narratives for each MLTC population segment; and 2) co-define systems visions, targets and potential solutions for each segment – prioritised through PPIE. Our development work will deliver a MLTC learning systems framework and strategic case for the Hub, with partners investing in long term iterative co-innovation for better MLTC prevention, care and system monitoring. This framework will drive prompt acceptability and feasibility testing for new technologies, services and pathways in useful contexts. For example, a novel technology to detect accelerating frailty from an ambient sensing device in the home would be validated within a whole pathway/system context of how the NHS and social care can respond to signals from the device and patient, and not an isolated validation of e.g. ability to sense activities of daily living.

### Design principles and systems science

**Figure 1.**
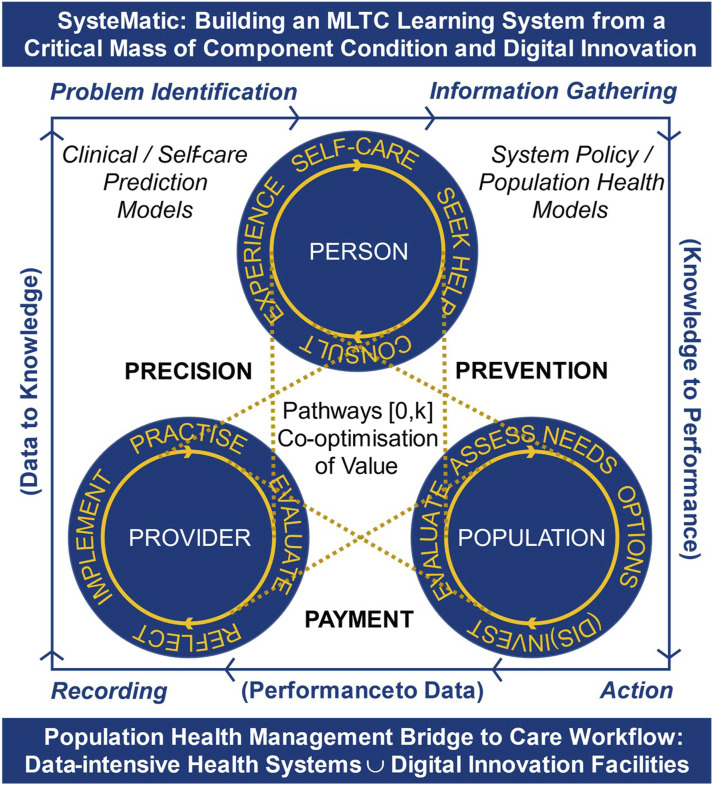
Schematic of design principles and systems science.

Our innovation hub design embeds a novel Triple Learning Health System framework as illustrated (Figure 1). We adopt human-centred^16,17^, inclusive^18,19^, equitable and participatory^20-22^ design principles to ensure an innately patient-centred, equitable and innovative approach. Design futuring^23,24^ and speculative design^25-27^ provide ways to research and build shared future MLTC scenarios that can help to identify systems innovation and transition targets and help to inform and incorporate the new technologies and tools.

Our cooperative of disciplines includes systems modelling, systems engineering, systemic design, clinical research, public health research, and health and care commissioning and provision.^14,15^ We apply three orders of systems science to health and care systems, distinguished by: a) how they define a system; b) the associated research methods; and c) the tools, frameworks and techniques. These three orders are:

1) 1st-Order optimises discrete components with clear boundaries, quantified inputs/outputs, crude ontologies and a governing process with feedback and error correction.^28-30^ For example, feedback to GPs on their potentially harmful prescribing of antipsychotics and interacting drugs with no wider context.
2) 2nd-Order is the mainstay of systems engineering in healthcare.^12,13^ Modelling is ontological, but tolerates ill-defined boundaries, uncertainty and unknowns, and may adopt simulation methods,^31-33^ or statistical process control.^34^ For example, modelling the potential impacts of a weight management clinic/service for people taking antipsychotic medication, after psychotic symptoms are controlled.
3) 3rd-Order extends to soft systems^14,35^ with porous boundaries, nonlinear behaviours, and contextual sensitivity.^15,36^ Key approaches are participatory; PPIE with practitioner co-design, action research,^37^ systemic design,^38-40^ and transition design.^41,42^ This approach suits transitioning from single condition/pathway/service optimisation,^43^ to integrated MLTC approaches.^44^ For example, transitioning from NHS weight-management referral to a package of wider cardiovascular risk reduction and social support/prescribing for people taking antipsychotic medication.

### Design cooperative approach to multiple long-term conditions

The complexity of MLTCs can be overwhelming unless research questions are focused. To avoid this risk, we will introduce MLTC scenarios in population segments in Stage 1, seeding co-produced research priority roadmaps in Stage 3. The Children & Families segment focuses on families under stress (parents and children) due to factors such as childhood adversity,^45^ neurodevelopmental disorders (Attention Deficit Hyperactivity Disorder/Autistic Spectrum Disorder, intellectual disability), other physical health issues (e.g. asthma) and positive and negative risk-behaviours (including sport/accidental trauma, sedentary lifestyle, self-harm, and substance use).^46^ The Working Life segment considers mid-to-late adulthood (∼30–60 years) with a focus on mental-physical health interactions in chronic pain/fatigue, mood disorders and risk factor accumulation (cardiovascular risk, metabolic syndrome/diabetes). The Pre-Frailty segment reflects mid-life through retirement (∼50–70 years)^47^ with a focus on multiple organ disorder, decompensation/failure of adapted psychosocial mechanisms (early retirement, life-transitions, suicide risk), hypothalamic-pituitary-gonadal axes (menopause), cognition and neurodegeneration (dementia) and musculoskeletal decline – with commonly missed opportunities to prevent frailty among people living with MLTCs. PPIE-prioritised solutions may include ambient sensing of sleep, heart rate variability and mood changes, hastening intervention to avoid frailty.

### Design cooperative 5-stage process to prepare the systematic innovation hub

#### Stage 0: Involves convening a design co-operative to ensure

A. **People Insights** will be achieved through putting a diversity of patients, practitioners, and public partners – including those seldom heard, at the centre of design. The group will consolidate lived experiences into person, journey and system maps, informing system failure scenarios.
B. **Health and Care Intelligence** will feed practitioners’ knowledge of MLTC segments into system maps and failure scenarios, using local data to quantify MLTC pressures and trajectories. The group brings clinical, social care, managerial and public health professionals together with public advisors, systematically grounded with inputs from the People Insights group.
C. **Systems Futures** will synthesise evidence for MLTC systems methodology and apply this to optimising existing services, driving innovation, and transitioning MLTC services to sustainable implementation of the right innovations. The group will draw upon substantial clusters of HealthTech industry partnerships and linked PPIE shaping new technologies and tools in Liverpool and Glasgow.

Deliverable: MTLC insight and design community ready to execute Stages 1–4.

#### Stage 1: Deep dives into MLTC epidemiology, experiences and services in 3 population segments

Questions: What are the MLTC burdens in our populations? What do people with MLTCs and practitioners see as systems failures and priorities? Route to answers: triangulation of lived experience, practitioner and data insights.

Using cultural probes^48^ and photovoice autoethnography,^49^ contextual interviews and generative workshops, the People Insights group will map experienced care pathways and system failure scenarios – identifying commonalities and differences in stakeholders’ perspectives. The Systems Futures group will consider the literature on healthcare systems design, extended to include social care and population health management; by targeted review of this diverse literature, we will identify patterns across theory and applied work to provide a preliminary ontology of principles, methods and techniques^50^. The Health Intelligence group will work with regional data teams to develop sociodemographic and MLTC profiles for the segments. This information may also help target neighbourhood initiatives to support families with complex lives including drug and alcohol problems.

Deliverables: 1) Ontology of MLTC systems principles and techniques; 2) MLTC population segment profiles; 3) MLTC journey maps in each segment; 4) system map of pathways and their bottlenecks for patients and practitioners; 5) system failure scenarios in and across segments.

#### Stage 2: Co-identify Priority MLTC systems failures and targets for action

We will define and prioritise the system pressures, maps, conditions, and failure points in collaboration with patient partners and healthcare professionals. The People Insights group will use the rich qualitative data and segment profiles from Stage 1 to populate arrays of system failure points. These in turn will be used to co-create MLTC (systems) research priorities and roadmaps.

Deliverables: 1) Shared problem definitions/priorities, system maps for each MLTC population segment linked to outputs from Systems Futures and Health and Care Intelligence that emphasises opportunities for optimisation, innovation and transition.

#### Stage 3: Co-define research, technology and intervention priorities for systematic

**Figure 2.**
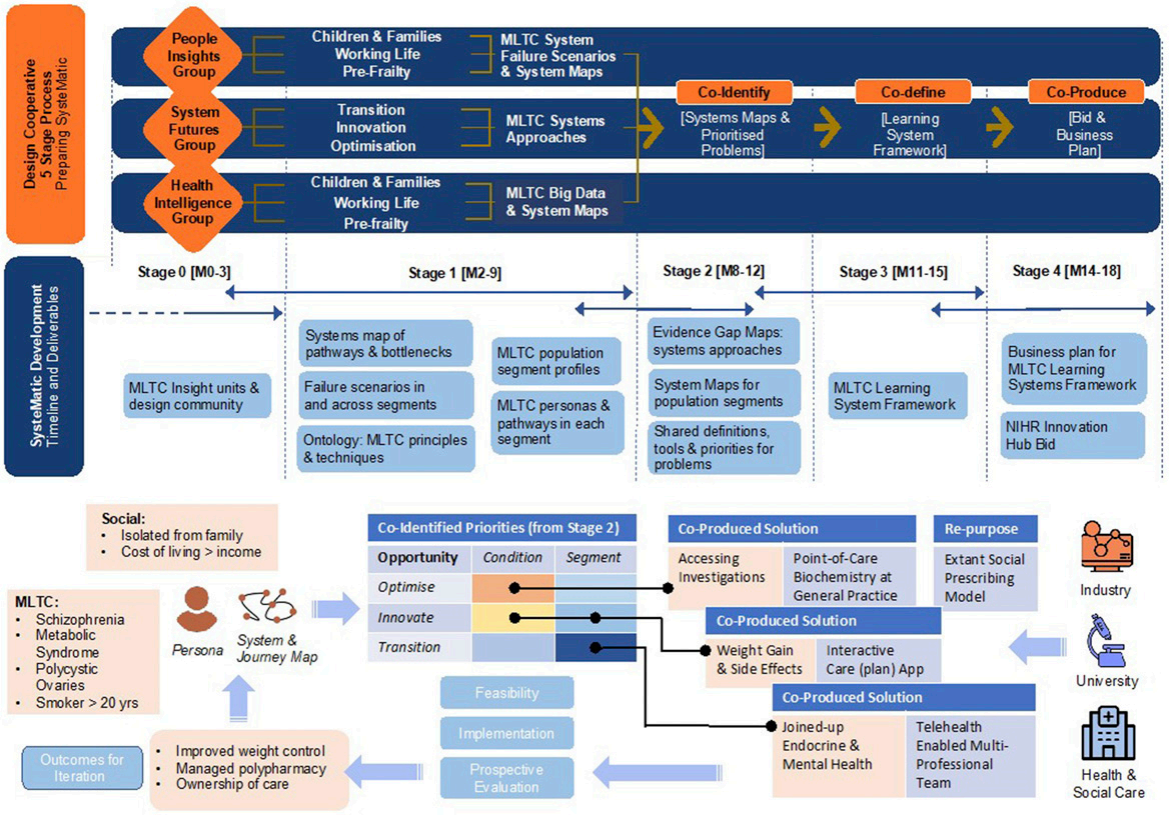
MLTC learning system framework.

The 3 working groups will iterate through workshops to generate a consensus on the key research, technologies and intervention targets needed to initiate a ‘learning systems flywheel’ of MLTC innovation, acknowledging that transformations never happen outright but rather through consistent efforts, building momentum until a major advance is achieved.^51^ PPIE groups will consider MLTC person maps and mock-ups of (systems) solutions for better experiences (Figure 2).

Deliverable: MLTC learning system framework with actionable examples in each population segment

#### Stage 4: Co-producing the SysteMatic MLTC innovation hub strategic plan with system partners

Stakeholders will co-produce a strategic plan to implement the MLTC learning system framework in the SysteMatic hub across Liverpool and Glasgow, with co-investment from academic health system and industry partners. A sandpit event, advertised through our networks, will gather working group members, public, and interested parties. The sandpit will spawn sub-groups responsible for each part of the plan.

Deliverables: 1) Strategic plan for MLTC action research and design as core business of two health & care systems in England and Scotland; 2) bid for National Institute for Health and Care Research (NIHR) SysteMatic MLTC Innovation Hub.

### MLTC systems knowledge commons

Outputs from Stages 1–4 will be organised, shared and disseminated online via an open knowledge commons similar to the Turing Commons.^52^ Outputs will be available to other Hub developers to encourage synergy and avoid repetition across the themes/projects, maximising public return on the funding for the development stage work. The outputs will also create a platform for the SysteMatic development team to align with UK strategic priorities (e.g. the UK Life Sciences Missions, NIHR priorities, UK Research and Innovation (UKRI) healthcare technologies and Department of Health and Social Care (DHSC) areas of research interest), and to collaborate with other institutions developing related hubs. Our findings will also be disseminated at public meetings and national and international events, through our online open knowledge commons and publication in peer-reviewed journals.

This study was approved by the University of Liverpool, Faculty of Science and Engineering Research Ethics Committee (approval number 12724) on August 22, 2023 and the University of Glasgow, College of Medicine, Veterinary and Life Sciences Ethics Committee (approval number 200230002) on October 6, 2023. We have an External Advisory Board to inform the strategic direction of the hub.

## Discussion

We have described an ambitious programme of research that aims to underpin development of an innovation hub (SysteMatic) designed to foster and implement solutions for people with MLTCs. This programme of research has integrated PPIE throughout the development phase. Our PPIE groups contributed to the decision to focus on three specific population segments, emphasising the importance of families with complex lives. They advised on terminology and methods throughout and have worked with the research team to craft the PPIE strategy. To maximise the inclusion of relevant lived experiences and specialist expertise, we will use a participatory, inclusive and human-centred design methodology pioneered by the Inclusionaries Lab.^53,54,57,58^

### Risks, barriers and mitigations

Such ambitious programmes always have potential risks. Two key risks relate to recruitment challenges, especially of “seldom heard voices” and failure to develop shared understandings and overcome interdisciplinary barriers. These risks have been mitigated through: our existing partnerships and infrastructure, for example, working with the Liverpool Civic Data Cooperative^55^ and the Byres Community Hub, Glasgow^56^ to ensure access to people with MLTCs from diverse backgrounds; and the incorporation of a research triangulation approach with iterative feedback to stakeholders from different disciplines, for maximum validity and reliability and development of a glossary of terms to promote understanding of disciplinary differences in terminology.

## Conclusion

While our key deliverable will be a strategic plan and funding application for an equity focused innovation hub (SysteMatic) for people with MLTCs, there will also be considerable learning and consolidation of sense-making tools based on our engagement work with the three groups of people with MLTCs, who will provide new insights into the lived experience of people with MLTCs and the key challenges they face in navigating current health and social care systems.

## References

[1] Skou ST Mair FS Fortin M , et al. Multimorbidity. Nat Rev Dis Primers 2022; 8(1):48. 35835758 10.1038/s41572-022-00376-4 PMC7613517

[2] The Academy of Medical Sciences . Multimorbidity: a priority for global health research. Report, London, UK, 20-21 June 2018.

[3] Mair FS May CR . Thinking about the burden of treatment. BMJ 2014; 349:g6680. 25385748 10.1136/bmj.g6680

[4] Makovski TT Schmitz S Zeegers MP , et al. Multimorbidity and quality of life: Systematic literature review and meta-analysis. Ageing Res Rev. Epub ahead of print 30 April 2019. DOI: 10.1016/j.arr.2019.04.005. 31048032

[5] Jani BD Hanlon P Nicholl BI , et al. Relationship between multimorbidity, demographic factors and mortality: findings from the UK Biobank cohort. BMC Med 2019; 17(1):74. 30967141 10.1186/s12916-019-1305-x PMC6456941

[6] Barnett K Mercer SW Norbury M , et al. Epidemiology of multimorbidity and implications for health care, research, and medical education: a cross-sectional study. Lancet. Epub ahead of print 10 May 2012. DOI: 10.1016/S0140-6736(12)60240-2. 22579043

[7] Thornicroft G . Physical health disparities and mental illness: the scandal of premature mortality. Br J Psychiatry 2011; 199(6):441-442. 22130744 10.1192/bjp.bp.111.092718

[8] Kivimäki M Batty GD Pentti J , et al. Association between socioeconomic status and the development of mental and physical health conditions in adulthood: a multi-cohort study. Lancet Public Health. Epub ahead of print 31 January 2020. DOI: 10.1016/S2468-2667(19)30248-8. 32007134

[9] Osborn DP Levy G Nazareth I , et al. Relative risk of cardiovascular and cancer mortality in people with severe mental illness from the United Kingdom's General Practice Research Database. Arch Gen Psychiatry 2007; 64(2):242-249. 17283292 10.1001/archpsyc.64.2.242

[10] Lennon MR Bouamrane MM Devlin AM , et al. Readiness for Delivering Digital Health at Scale: Lessons From a Longitudinal Qualitative Evaluation of a National Digital Health Innovation Program in the United Kingdom. J Med Internet Res 2017; 9(2):e42. 10.2196/jmir.6900 PMC5334516 28209558

[11] Cresswell K Sheikh A . Organizational issues in the implementation and adoption of health information technology innovations: an interpretative review. Int J Med Inform 2013; 82(5):e73-86. 23146626 10.1016/j.ijmedinf.2012.10.007

[12] John Clarkson P . What has engineering design to say about healthcare improvement? Design Science 2018; 4:e17.

[13] Royal Academy of Engineering . Engineering better care: a systems approach to health and care design and continuous improvement. Report, Royal Academy of Engineering, London, UK. August 2017.

[14] Checkland P. Soft systems methodology: a thirty-year retrospective. Syst Res Behav Sci 2000; 17(S1):S11-S58.

[15] Preiser R . Identifying general trends and patterns in complex systems research: An overview of theoretical and practical implications. Syst Res Behav Sci 2019; 36:706–714.

[16] Bazzano AN Martin J Hicks E , et al. Human-centred design in global health: A scoping review of applications and contexts. PLoS One 2017; 12(11):e0186744. 29091935 10.1371/journal.pone.0186744 PMC5665524

[17] Leason I Nickpour F . The state of inclusive and human-centred design in oral healthcare. In: DRS2022 (eds Lockton D Lenzi S Hekkert P Oak A Sádaba J Lloyd P ), Bilbao, Spain, 25 June - 3 July 2022. DOI:10.21606/drs.2022.698.

[18] Lim Y Giacomin J Nickpour F . What Is Psychosocially Inclusive Design? A Definition with Constructs. The Design Journal 2020; 24(1):5–28.

[19] Clarkson PJ Keates S Coleman R Lebbon C . Inclusive design: Design for the whole population. London: Springer, 2003, p1-307.

[20] Spinuzzi C. The methodology of participatory design. Technical communication 2005; 52(2):163-174.

[21] Vandekerckhove P de Mul M Bramer WM , et al. Generative Participatory Design Methodology to Develop Electronic Health Interventions: Systematic Literature Review. J Med Internet Res 2020; 22(4):e13780. 32338617 10.2196/13780 PMC7215492

[22] Pilemalm S Timpka T . Third generation participatory design in health informatics--making user participation applicable to large-scale information system projects. J Biomed Inform. Epub ahead of print 1 October 2007. DOI: 10.1016/j.jbi.2007.09.004. 17981514

[23] Fry T . Design futuring: sustainability, ethics, and new practice. Sydney: University of New South Wales Press, 2009, p.71.

[24] Sandjar K , et al. Expanding modes of reflection in design futuring. In: CHI Conference on Human Factors in Computing Systems, New York, USA, 25-30 April 2020, 1–15. New York CHI: Conference on Human Factors in Computing Systems.

[25] Dunne A Raby F . Speculative everything: design, fiction, and social dreaming. Cambridge, Massachusetts: The MIT Press, 2013 pp1-240.

[26] Auger J. Speculative design: crafting the speculation. Digital Creativity 2013; 24(1):11–35.

[27] Galloway A Caudwell C . Speculative design as research method: From answers to questions and “staying with the trouble. In: Galloway A Undesign Caudwell C (eds) Undesign. 1st ed.. Routledge, 2018, pp. 85–96.

[28] Garnett GP Cousens S Hallett TB , et al. Mathematical models in the evaluation of health programmes. Lancet. Epub ahead of print 8 April 2011. DOI: 10.1016/S0140-6736(10)61505-X. 21481448

[29] Griffiths JD Knight V Komenda I . Bed management in a Critical Care Unit, IMA J Manag Math 2013; 24(2):137–153.

[30] Calder M Craig C Culley D , et al. Computational modelling for decision-making: where, why, what, who and how. R Soc Open Sci 2018; 5(6):172096. 30110442 10.1098/rsos.172096 PMC6030334

[31] Vázquez-Serrano JI Peimbert-García RE Cárdenas-Barrón LE . Discrete-Event Simulation Modeling in Healthcare: A Comprehensive Review. Int J Environ Res Public Health 2021;18(22):12262. 34832016 10.3390/ijerph182212262 PMC8625660

[32] Sterman J Oliva R Linderman K , et al. System dynamics perspectives and modeling opportunities for research in operations management. Journal of Operations Management 2015; 39–40:1-5.

[33] Cassidy R Singh NS Schiratti PR , et al. Mathematical modelling for health systems research: a systematic review of system dynamics and agent-based models. BMC Health Serv Res 2019; 19(1):845. 31739783 10.1186/s12913-019-4627-7 PMC6862817

[34] Benneyan JC Lloyd RC Plsek PE . Statistical process control as a tool for research and healthcare improvement. Qual Saf Health Care 2003; 12(6):458-464. 14645763 10.1136/qhc.12.6.458 PMC1758030

[35] da Costa J Diehl J Snelders D . A framework for a systems design approach to complex societal problems. Design Science 2019; 5.

[36] Checkland P . Four Conditions for Serious Systems Thinking and Action. Syst Res 2012; 29: 465-469.

[37] Koshy E Koshy V Waterman H . Action research in healthcare. Sage, 2010, pp1-145.

[38] Jones P Kyoichi K . Systemic Design Theory, Methods, and Practice: Theory, Methods, and Practice 2018; DOI:10.1007/978-4-431-55639-8.

[39] Jones P . Systemic Design: Design for complex, social, and sociotechnical systems. In: Metcalf G.S. Kijima K. Deguchi H. (eds) Handbook of Systems Sciences. Springer, Singapore 2020. DOI:10.1007/978-981-13-0370-8_60-1.

[40] Buchanan R . Design Research and the New Learning. Design Issues 2001; 17(4): 3–23.

[41] Irwin T . Transition Design: A Proposal for a New Area of Design Practice, Study, and Research Design and Culture 2015; 7(2):229–246.

[42] Bisson M Palmieri S Ianniello A et al. Transition design: an opportunity for design and designers. In:16th International Technology, Education and Development Conference (eds Gómez Chova L López Martínez A Candel Torres I ), virtual conference, 7-8 March 2022, pp2692-2702. INTED2022.

[43] Augustsson H Churruca K Braithwaite J . Re-energising the way we manage change in healthcare: the case for soft systems methodology and its application to evidence-based practice. BMC Health Serv Res 2019; 19(1):666. 31521156 10.1186/s12913-019-4508-0 PMC6744652

[44] Ainsworth J Buchan I . Combining Health Data Uses to Ignite Health System Learning. Methods Inf Med 2015; 54(6):479-487. 26395036 10.3414/ME15-01-0064

[45] Neufeld SAS . The burden of young people's mental health conditions in Europe: No cause for complacency. Lancet Reg Health Eur 2022; 16:100364. 35345644 10.1016/j.lanepe.2022.100364 PMC8956936

[46] Armocida B Monasta L Sawyer S et al. Burden of non-communicable diseases among adolescents aged 10-24 years in the EU, 1990-2019: a systematic analysis of the Global Burden of Diseases Study 2019. Lancet Child Adolesc Health 2022; 6(6):367-383. 35339209 10.1016/S2352-4642(22)00073-6 PMC9090900

[47] Chang AY Skirbekk VF Tyrovolas S , et al. Measuring population ageing: an analysis of the Global Burden of Disease Study 2017. Lancet Public Health 2019; 4(3):e159-e167. 30851869 10.1016/S2468-2667(19)30019-2 PMC6472541

[48] Wherton J Sugarhood P Procter R , et al. Designing assisted living technologies 'in the wild': preliminary experiences with cultural probe methodology. BMC Med Res Methodol 2012; 12:188. 23256612 10.1186/1471-2288-12-188 PMC3552936

[49] Catalani C Minkler M . Photovoice: a review of the literature in health and public health. Health Educ Behav 2010; 37(3):424-451. 19797541 10.1177/1090198109342084

[50] da Costa Junior J Diehl JC Snelders D . A framework for a systems design approach to complex societal problems. Design Science 2019; 5:e2.

[51] Collins JC . Turning the Flywheel: A Monograph to Accompany Good to Great. London: Harper Business, 2019, pp1-46.

[52] The Alan Turning Institute . The Turing Commons, www.turing.ac.uk/research/research-projects/turing-commons (2024, accessed 27 June 2024).

[53] Shaw C Nickpour F . Embedding Narratives through Inclusive Design: A Multidisciplinary Exploration of the Roles and Applications of End-User Narratives Within and Beyond a MedTech Design Process. The International Journal of Designed Objects 2024; 18(2):1-18.

[54] Shaw C Nickpour F . Embedding and embodying narratives in the collaborative development of life-changing healthcare technologies. In*:* IASDR 2023: Life-Changing Design (eds De Sainz Molestina D Galluzzo L Rizzo F Spallazzo D ), Milan, Italy, 9-13 October.

[55] Civic Data Cooperative. https://civicdatacooperative.com (accessed 28 June 2024).

[56] Byres Community Hub , School of Health and Wellbeing, University of Glasgow. www.gla.ac.uk/schools/healthwellbeing/byrescommunityhub/about/ (accessed 28 June 2024).

[57] Leason I Longridge N Mathur MR Nickpour F . An opportunity for inclusive and human-centred design. Br Dent J 2022; 233(8):607-612. 36307697 10.1038/s41415-022-5101-1

[58] Leason I Longridge N Nickpour F . Application and evolution of design in oral health: a systematic mapping study with an interactive evidence map. Community Dent Oral Epidemiol 2024; 52(1):1-12. 37526262 10.1111/cdoe.12892 PMC10952138

